# Love versus/and/as politics in Miroslav Krleža’s *Banquet in Blitva *and Virginia Woolf’s *Between the Acts*

**DOI:** 10.12688/openreseurope.20783.1

**Published:** 2025-07-23

**Authors:** Lada Čale Feldman

**Affiliations:** 1 Faculty of Humanities and Social Sciences, University of Zagreb, Zagreb, 10000, Croatia

**Keywords:** Virginia Woolf, Between the Acts, Miroslav Krleža, Banquet in Blithuania, gender politics

## Abstract

When asked by literary historian Predrag Matvejević to express his views on the eventual dramatization of his novel *Banquet in Blitva* (the first two books of the novel were published in 1938–39, the third one in 1962, while the English translation of the first two books appeared in 2004), the Croatian writer Miroslav Krleža – as if to spite the predominant interpretive lenses through which the novel is read in the critical mainstream (cfr. Malić 1963, Vidan 1964, Biti 1984, Flaker 1993, Žmegač 2001, and Kovačević 2013) – insisted that the dramaturgical bone of such an endeavour should be “the love theme” joining Karin Michelson to the protagonist Niels Nielsen, and not “the officers, revolt, army” and “state parades,” which should remain sheer “decorative motives” (Matvejević, 1987: 207). The paper examines the implications of this claim by placing the analysis of the novel within a double – conceptual and comparative – framework: the first one will permit us to explore Krleža’s novel’s “grammar of motives,” as proposed by K. Burke’s dramatistic model (cfr. Burke, 1945), while the second will return to the already detected formal, thematic and contextual correspondences between the Croatian novel and Virginia Woolf’s posthumously published *Between the Acts* (cfr. Čale Feldman, 2008), in order to address the opposing ways in which the two novels deal with gender politics as a neglected aspect of the rationale of WWII.

Literature is no one’s private ground; literature is common ground. It is not cut up into nations; there are no wars there. Let us trespass freely and fearlessly and find our own way for ourselves. It is thus that literature will survive this war and cross the gulf – if commoners and outsiders like ourselves make that country our own country, if we teach ourselves how to read and to write, how to preserve and how to create.
  Virginia Woolf, *The Leaning Tower*
  To be critical, to fully exercise critique in literary criticism, would mean in this domain several things simultaneously. (...) It would mean a dialectical critique that attuned itself to the dialectics in the world and the dialectics in the work – according to the work of art dialecticity of its own. It would mean addressing the specific techniques of critique proper to and figured in aesthetic presentation, which necessarily operates through an entirely different grammar than critique in Karl Marx’s *Critique of Political Economy* or Gayatri Chakravorty Spivak’s *Critique of Postcolonial Reason*, the way in which aesthetic mediation necessarily works figuratively, obliquely, syncretically. Literature deserves a dialectical theory of its own dialecticity.
  Anna Kornbluh, *We Have Never Been Critical: Toward the Novel as Critique*

In what follows, I propose an eventual ‘reassessment’ of two important works of fiction that undoubtedly belong to the canon of the 20 ^th^ century political novel: Miroslav Krleža’s *The Banquet in Blitva* ^1^ and Virginia Woolf’s *Between the Acts* ^2^. My aim is to show that the two novels have some striking similarities in terms of interests, motives and strategies, although they seem to be far removed from each other, as the first is set in an imaginary country in north-central Europe, riven by poverty, crime, ignorance, thefts and killings, if not mass murders, espionage and political opportunism – resembling in many humorous details the Kingdom of Yugoslavia of the late 1920s and 30s – while the second is set in contemporary imperial England, in an estate, named Pointz Hall, characterised by wealth, tradition and aristocratic privilege, a prototypical seat of patriarchy. The main compositional technique that brings these novels closer together is the use of internal theatre frames and theatrical metaphors to engage in self-reflexivity and to explore the ontological, epistemological and performative limits of novel-writing in the context of an impending war. Although both Krleža and Woolf are rather pessimistic about the prospect of this catastrophe and the efficacy of their literary visions, both ironically frame the action of their novels as comedy: while Krleža begins his story with the emphatic proclamation “Incipit comoedia Blithuanica!”, Woolf’s inserted play – which features Queen Elizabeth, among others, but is performed by obscure, ordinary villagers – draws on fragments of Elizabethan and Restoration comedy to reflect the public’s stupidity and blindness to the real causes of war. It should also be noted that both novels insert the contemporaneity they deal with into a historical span that begins with primordial mud – a word that is explicitly and repeatedly invoked in *Between the Acts*, and a word which Krleža compulsively returns to throughout his entire work as a primary emblem of his home country’s slavish backwardness, but even more forcefully in *Banquet in Blithuania*, since in Croatian, where “mud” sounds *blato*, the word forms an assonantal chain together with *Blitva* and *Blatvija*, original names of the two neighbouring states in which the action of the novel takes place.

I must immediately add that the implications of this double reassessment, which mainly concerns the lens of gender, could affect the interpretative fate of Miroslav Krleža’s novel much more than that of Virginia Woolf’s *Between the Acts*, for obvious reasons to do with the lack of gendered readings in Krleža’s case, and, of course, with the incommensurability of the critical response to the two authors. This is to some extent anticipated by these novels themselves, through their explicit addressing of the cultural power dynamics, that is, the imbalance between centre and periphery, or between the sexes, that determines artistic production and reception, but given the worldwide fame Woolf has achieved over the last fifty years as a feminist icon, and the canonization of her posthumously published novel in Gilbert and Gubar’s *No Man’s Land* as the rightful culmination of Woolf’s work and one of her most poignant anti-war novels ( 3 on page 3–56), the likelihood of offering a significant re-reading of Krleža’s novel from a gender perspective definitely seems much greater. This is also why Virginia Woolf’s novel appears here primarily as a comparative yet to a certain extent also contrasting approach to questions of national and gender identity and the ways in which the fiction of the former is supported or undermined, as it were, by the flesh of the latter, such as roles in a performance through the bodies and minds of both brilliant and inept actors or, as we shall see in Krleža’s case, puppets.

Like *Between the Acts*, the three-volume *Banquet in Blithuania* was written after the experiences of the Spanish Civil War, on the eve of Hitler’s invasion and the outbreak of the Second World War. The first two books were published in 1938–39. The third book, although already partly written at the time, was not published until 1962 and has unfortunately not been translated into English ^3^, with the dubious explanation of the translators of the first two books, Edward Dennis Goy and Jasna Levinger-Goy, that “the first two volumes form a unit and can be offered as such” ( 4 on page 337). Krleža would have disagreed, since he insisted that “with the third volume of the novel” the story “comes to a conclusion as a compositional whole, in the form of a political and moral catharsis of the main character, who has realised that isolated individual resistance in the fight against injustice and lies ends in romantic ruthlessness” (both quotes can be found in: 6 on page 508–510). Together with the author’s five-volume novel *Banners*, *Banquet in Blithuania* is regarded by Croatian critics as a mixture of, on the one hand, the originally conceived political pamphlet against the actual phenomena of political, moral and aesthetic tyranny that plagued both Eastern and Western Europe of his time, and, on the other hand, a psychological novel that traces the intimate dilemmas of the protagonist and thus, at least in Zdravko Malić’s opinion, partially exceeds the qualification of “the political novel” ( 7 on page 171). It is also one of Krleža’s most experimental works: although critics more or less agree on its genre affiliation, they differ in their attempts to determine the modernity of its style and structure, most of them recognising its supposed “programmatic objectivism,” characteristic of all Krleža’s writings in the 1930s, if not, in Vladimir Biti’s words, a “strong hand” of a “preachy narrator,” typical of what this critic calls “cautionary literature” ( 8 on page 67). Although the novel is populated by a multitude of characters – state officials, police agents, spies and informants, cardinals, peasants, poets, painters, sculptors, doctors, professors, historiographers and political rebels – a good number of whom get the chance to elaborately present their opposing views on the Blithuanian world, critics claim that, on the whole, one can grasp its “unified image,” even if it is the image of a “madhouse” ( 9) – a word shared by most of the novel’s speakers as a description of Blithuania – for the narrator’s demiurgic position remains unchallenged: he appears, the critical consensus goes, as the director of a performance, which he seems to view from a distance, both geographically, as he is from the start located somewhere in a “dim and distant foreign country” ( 1 on page 5), and chronologically, as in the third book he reports on this “performance” two or three decades after its, as he also initially formulates, “operatic” course ( 1 on page 7).

 Now, this assumption regarding the narrator’s authority hardly corresponds, in my view, to the polyphonic intricacies of the novel, which, although largely written in free indirect speech, abounds in theatrical analogies that destabilise any recognisable narrative stronghold and prevent us from clearly deciding whose perspective to adopt. After all, performances are never fully controlled by their directors: as *Between the Acts* also attests, the actors improvise, reality intervenes in unpredictable ways, and the audiences ascribe a whole series of their own associations to what they experience. It becomes hard to tell “who speaks,” for both novels create what Christine Froula, speaking about Woolf’s novel, describes as “a virtuoso interplay of utterance, conversation, silent reflection and communication, reported speech, quotation, the written word in newspapers, script and performance,” whereby “the voice of an omniscient, moralising narrator is exchanged for a kind of metavoice that belongs to no one” ( 10 on page 302, 304). Interestingly, it is precisely such a metavoice that is implicitly referred to at one point as belonging to “the Invisible Director” in the *Banquet in Blithuania* ( 1 on page 326). This, in turn, makes the politics of both novels a somewhat shaky terrain. In the case of the *Banquet*, there is no doubt that the ultimate resolution of the ‘Blithuanian question down the centuries’ is the main theme and political stake around which the novel organises its narrative material, juxtaposing in this context its two main antagonists, the murderous usurper and tyrant, the Lord Protector of Blithuania, Colonel Christian Barutanski, and on the other side, his former school friend, the humanist, lawyer and publicist Niels Nielsen, who resigns his commission in Barutanski’s elite military squad, returns all his medals and becomes a publicist challenging the dictatorship.

However, the novel is not a “sermon” on the truthfulness, dangers and pitfalls of their respective political visions of Blithuania’s history and reality, but a “dramatised” clash of their – and not only their – competing political discourses. In his “dramatistic” analysis of language as symbolic action, Kenneth Burke considers such explanatory discourses as “casuistries” and analyses their different ways of ascribing motives to human action ( 11 on page xi). His *Grammar of motives* begins, interestingly, with the motto *Ad bellum purificandum*, for in the late 1930s this theorist became interested in the possibility of bridging conflicting ideologies and formulating their dialectical “common ground,” the logical substance that is their causal ancestor. He found it in the ever-changing interplay of his five key dramatistic concepts of **act**, **scene**, **agent**, **action** and **purpose**, to which I will return in a moment. For Woolf, as for Krleža, this common ground, as we could have gathered from the motto of this discussion, is definitely to be found in literature, the kind of literature that Burke also favoured: a literature that would counter the “false cooperation” brought about by war and the monological unity of modern dictatorship with a diversity of voices and a dialectical unity of “true cooperation”. That is why he conceived his own work as a rhetorical/aesthetic project that would “transcend physical war with verbal combat and pacification, turning its tragic absolute into the **comic** potentiality of alternate perspectives” ( 12 on page 286–302).

I refer to Burke’s *Grammar of Motives* for even more precise reasons, because the question of motive, both in its psychosocial and literary-theoretical sense, emerges in a curious protest against any simple political reading of his novel that Krleža himself expressed when he was asked by a respected critic and intellectual, Predrag Matvejević, to share his views on a possible dramatisation, i.e. an adaptation of *Banquet in Blithuania* for the stage:

The Nielsen-Barutanski relationship is not as important as the relationship between Nielsen and Karina. Nielsen’s love for Karina is one of the main motives in *The Banquet*. A man who fights for some basic principles, let us call them humanistic or democratic in the broader sense of the word, actually becomes the murderer of his own love. Nielsen kills Karina. And not just Karina, he actually kills a whole series of people, not just Barutanski or Rajevski or Larsen, and I do not know who else, it’s five or six, it’s Georgis and Kerinis and co. They will all go down in history, if you like, as victims of Nielsen himself. (...) So I would start with the Karina-Nielsen love motive, with her diary. It’s a story of their mutual affection. She does not dare to confess and admit that she was or is in Barutanski’s service, in whose entourage there is not a single officer who has not slept with her. My God, her husband was killed, she is a young woman left alone, they have taken this young woman as a kind of flesh, they have used her as young flesh. (...) The crisis of her love, that finds constant expression in her diary, is thus the main motive of the novel. Nielsen falls victim to intrigue when he is shown a document she gave them to read, the concept of his speech before it was published; implying that she was in their service, *which need not be the truth* (emphasis LČF). And then their final conflict in the novel erupts when Nielsen no longer wants to speak to Karina, they break up and Karina commits suicide to prove to him that she is innocent: and he becomes the murderer.
  And everything else: Officers, revolt, army, the so-called state parades, Rajevski and the Academy and the triumph of Barutanski, everything else are decorative motives that can speak and mean like on TV. The *Banque*t is in fact a psychological drama: Nielsen is guilty of Georgis, whom he kills himself, but he is also guilty of the murder of Karina, whom he kills by proxy, and then of Barutanski and the other young officer, whose name I can not remember; there are about ten or fifteen dead bodies, over whose corpses he walks in a Shakespearean fashion at the end. He is a murderer like Richard III and holds up a banner of his higher convictions. (...) There is another character in this novel who is no less important: Blithauer. He is there as a kind of counterpoint, a kind of socialist counterpoint: he proves to Nielsen that he has no idea of the milieu in which he moves and that, after all, he has not strayed very far from Barutanski. ( 13 on page 205–207)

Lest anyone think that I am relying too much on intentional fallacy here, I would like to temper Krleža’s insistence on love as the main motive of the novel and its protagonist with two remarks. The first concerns the writer’s sly avoidance of the obvious, namely that of all the Shakespearean figures with whom one might associate Nielsen, it is not Richard III who fits Nielsen’s profile, but rather Hamlet, if one takes the constellation Krleža draws here seriously, with Karina as the undeservedly accused modern Ophelia, who is also a suicide, and a host of other corpses Hamlet produces during the course of Shakespeare’s play – first Polonius, then Rosencrantz and Guildenstern, then Laertus, Gertrude, and finally Claudius. The fact that the backbone of a possible dramatisation of the novel is a pre-existing play, containing itself a play-within-the-play, cannot surprise us, since in the entire novel such analogies appear at almost every narrative corner, not only through numerous references to the plays of Shakespeare, Racine, Molière or Ibsen, but also to novels containing theatrical frames such as *Don Quixote*, and to other, less specified theatrical terms such as comedy, stage, producer, masks, Italian opera, actor, clown, puppet and the like. All these allusions foreshadow and follow the central event of the novel, which takes place after Nielsen has fled from his would-be murderers in Blithuania to his country’s main adversary, Blatvia – namely his participation in the opening night of the Blatvian national theatre, where he attends a puppet performance, indeed a puppet play-within-the-play with a protagonist who is in many ways Nielsen’s double and is appropriately named Fortunato Yorick.

What I want to emphasise here is the compatibility of these obsessive associations with drama and theatre – which place all the characters’ actions on a questionable ontological footing and deprive their discursive versions of Blithuania’s political moment of any kind of firm perspectival hold – with Kenneth Burke’s dramatistic model with which this rhetorician analyses the semantics and grammar of all motivational discourse. Both Krleža’s and Woolf’s novels could be described as ‘dramatistic’ in the Burkean sense, for both use the theatrical frame to pinpoint and engage the fundamental tensions and ambiguities within the Burkean pentad – act, scene, agent, agency and purpose – whose different “ratios” determine the rhetoric, attitude and commitment of their characters towards the unfolding of history: materialistic, idealistic, pragmatic, realistic or mystical, depending on which concept they believe dominates the dialectic between the above-mentioned elements of human “drama” ( 11 on page 128). Thus, Krleža’s title itself seems to accentuate the dominance of the “scene” in various “ratios” that will come to light in the contrasting casuistries of the novel’s various characters. On the other hand, the title of Woolf’s novel – *Between the Acts* – emphasises Burke’s central concept of “act” most clearly in its constitutional capacity and polysemic scope and thus also alludes to Hamlet’s dilemma – to act or not to act – which, as we have already seen, underlies Nielsen’s ethical and political situation.

Furthermore, by using drama as their primary analogy, these novels draw our attention to both the historical accretion and the contingency of inherited patterns of behaviour – including gendered ones – and to the fact that the cultural scripts that people follow, sometimes as self-assured actors, sometimes as non-thinking puppets, derive from particular ideological stakes and interests. While deeply rooted in the human mind and behaviour, these blueprints can nevertheless be revised and changed, if only through words as symbolic action. From the Burkean perspective, to quote Robert Wess, “a new act can re-hierarchise old cultural priorities to establish new ones,” since “an act in its novelty is irreducible to its antecedent circumstances however deeply it is shaped by them. In this irreducibility resides the act’s constitutional capacity to go beyond its antecedent situation in transforming it” ( 14 on page 146). The same goes for long-established and perpetuated cultural scripts: “constituted in history, acts can always be agonistically melted down and reconstituted – constitutions are agonistic instruments” ( 14 on page 146). Consider the parallels in the following passages, in which the two novels seem to attribute the same maleficence and also the same constitutional power to the words:

Man finds comfort in words, and, beware: words, some nonsensical words, are made like vampires into metaphysical concepts, into independent, egoistic beings, so to speak, these stupid human words are made into something sacred, into gods, into political and popular shrines, into banners, into slogans, and, beware, these stupid words will one day take us to hell, because words without any meaning are nothing but hallucinatory chatter in a hallucinatory dream... ( 5 on page 41–42)
  Words this afternoon ceased to lie flat in the sentence. They rose, became menacing and shook their fists at you.
  Words raised themselves and became symbolical.
  Her words peppered the audience as with a shower of hard little stones.
  Words of one syllable sank down into the mud. She drowsed; she nodded. The mud became fertile. Words rose above the intolerably laden dumb oxen plodding through the mud. Words without meaning — wonderful words. ( 2 on page 59, 71, 78, 212)

One could argue that Krleža’s protagonist turns out to be a somewhat resigned writer who eventually consoles himself with “a box of lead letters” as his only worthy weapon, as Nielsen explicitly states in the very last – and, at least in Croatia, very famous – sentence of the novel, but it is highly significant that his metafictional counterpart in the inserted performance, Fortunato Yorick, asks the puppets around him to rebel against the imperative of the “string that controls their movements” ( 1 on page 327), as if pointing to the actual power of words in a performative context to evoke action. It is also no coincidence that Woolf’s last artistic figure, Miss La Trobe, is a playwright and director, someone who makes words turn into physical gestures, and thus seriously intends to intersect the symbolic with the history itself. In this respect, her ultimate confidence that there is always a new play waiting behind the one she has just staged, among other associations, betrays the hope that civilisation might change its violent course for the better under the beneficent pressure of an artistic intervention that deploys actual bodies and summons an actual audience. The fact that both Krleža’s and Woolf’s plays-within-a-novel and even Mousetrap-like plays-within-a-play-within-a-novel are threatened by a sudden intrusion of the Real – in the first case by the assassination of the Blatvian Prime Minister, in the second case by the thundering flight of twelve aeroplanes – in addition to denouncing the overwhelming presence of violence, only confirms the value that both authors derive precisely from the precarious nature of theatrical staging, from its embedding in the contingencies of historical reality.

My second remark, however, *à propos* Krleža’s commentary on the primacy of the love theme, concerns his blatant neglect in this passage of the fact that around eighty percent of his novel is still devoted to the dismissed “decorative motives”: it is therefore extremely difficult not to acknowledge the dominance of the masculine element in the novel, so much so that a lot of studies devoted to the *Banquet in Blithuania* completely disregard the motive of love and any mention of female characters. However, there are still a good deal of them – not only models of allegorical emblems of Blithuanian national glory, moulded into sculptures, “bronze Blithuanias kneeling like oriental slave girls,” appearing on stamps, coins, medals and flags; or singers and actresses, who sometimes also act as police informers and are therefore denounced by their admirers as whores who deserve to be killed; or anonymous wives of Barutanski’s dignitaries, who are regularly described as petty bourgeois parasites who benefit from all the privileges of their husband’s criminal business, but also some significant others of the two antagonists: their two wives, Barutanski’s Ingrid, a “bluestocking” and a “poetess,” and Nielsen’s Agnes, who, he strikingly recalls, was once dressed as a Carmelite nun during a carnival; then Nielsen’s mother, whose bed Barutanski once also shared when she hid him after his assassination of the old regime’s prime minister, Muzhikovski, and the two lovers – Barutanski’s little Solvejg, a distant echo of the safe haven of Ibsen’s Peer Gynt, and finally Karin Michelson, Nielsen’s fatal victim, as we have already heard, who dies by suicide very soon, near the end of the second book, but then reappears in the third volume, not only through inserted passages of her diary and her posthumous letters to Nielsen, but also through her constant presence in Niels Nielsen’s dreams of guilt.

It is precisely through the introduction of this characteristically surrealist framework that the aforementioned ontological dubiousness of the novel’s fragments is given an additional layer of theatricality, referring however to what Freud named the ‘other stage,’ the unconscious, whose actions appear to Niels Nielsen as being controlled by the same “invisible director” that had already signalled its presence at the end of the novel’s second book in the form of the acousmatic voice that announces and dominates the Blatvian opening performance, to the point of representing its “most concealed object” ( 15 on page 50–94), its enigmatic origin, the already mentioned unidentifiable “string” making puppets move. I insist on this connection, which unites the embedded play, dreams, and dealings in the novel’s external framework, because it structurally reflects Burke’s insistence on the ambiguity of human motivation, its dependence on both the extrinsic, social/material context of one’s action and the intrinsic factor, the pull of the psyche. As M. Elisabeth Weiser puts it, to ignore this ambiguity in the context of an impending war, meant for Burke, firstly, “to equate the intrinsic with the unique” and to ignore its “many similarities” with others, and secondly, to expose one’s own demands for change to a refutation that invokes one of the two determining motivators, ‘scene’ or ‘psyche’, in order to prevent any action ( 12 on page 296). This is exactly what Barutanski does when he alternately tries either to convince Nielsen of Blithuania’s irrevocable backwardness and corruption or to dismiss Nielsen as a political opponent by quoting his medical records, in which Nielsen is diagnosed as a psychopathological case. Therefore, it is necessary to address the ambiguities that arise from the clash of these different frames and levels in the novel. A Freudian reading here does not preclude reference to Burke’s concepts: Burke was a great admirer of Freud, and, like Lacan, treated Freud’s dream tropes and symptom formations as subsets of language as a whole, a kind of Freudian “grammar of motives,” a way of interpreting all kinds of human symbolic action which Burke considered to be in parallel with his own project ^16^.

Therefore, let us ask ourselves why Karin Michelson, who was practically non-existent in the first two volumes, suddenly became the driving force in the last book of the *Banquet*, the main character haunting the protagonist’s unconscious. She appears on the “other stage” in her various guises, not only in her former capacity as an unrealised pianist and enigmatic and talented Chopin fanatic, always shrouded in real and unreal mysterious veils; or a melancholic, obsessive visitor to churches for apparently cultural-historical reasons, always prone to mysticism, tears and sudden changes of mood, as well as to conversations about the so-called “ultimate questions”; a weak soul romantically enraptured by the prospect of a long couple she might form with Nielsen; a woman, as Nielsen complains, incapable of following “any kind of logic” ( 5 on page 244), incapable of understanding his crucial, indeed messianic role in Blithuanian history; but also as a specter denouncing her undeserved victimhood, a bleeding cat which, in his dreams, Nielsen carries in his suitcase, a porcelain doll now hanging from a rope, now blasted by Barutanski’s explosions, a figure seducing Nielsen in the theatre box by her borrowed appearance from other women he encountered, above all Gisele, Blithauer’s wife; a loyal companion willing to follow Nielsen on his oneiric suicidal falls from rooftops and towers, and finally, as a bitter commentator on this man’s entire civilising project, which to her is nothing other than a vacuous, shameful and infantile endeavour to live out his most basic instincts at the expense of millions upon millions of innocent others:

Women, who of course have no idea about anything, I beg your pardon, are nevertheless not created as blind and deaf creatures, and yes, I am sorry to have to say it, it is true, women are not gifted, because it is strange that not a single one has yet been born who could define what the whole appeal of all that is considered “masculine" actually lies in? “The man’s mind”, “the man’s character”, “the man’s criteria”, and, in general, all the masculine things you profess, as if you were always speaking from above, from a pedestal, as if all these masculine things could not be reduced to the little idiot from Brussels who, however much he is taken in by the importance of his water system, thinks he is particularly wise when he produces rain. I am certainly just a stupid woman, I rant and have no idea about anything, and I certainly can not be a philosopher in bed when you all always seem so immeasurably more intelligent than us... Yes, that’s what you all are, liquidators of all that is humanly weak, and you are so numerous that one can not even count how many of your kind there are. You are all out to repair heaven and earth, you great masters of the world, without even a trace of a heart in you! Man is just another guinea pig to you, but the human heart is not an anatomical apparatus, you forget that, gentlemen! ( 5 on page 253)

What does it mean to see in Nielsen’s love for such a woman – alluding here to what Freud considered to be one of man’s greatest early achievements that led to the emergence of civilization – not only the dramaturgical core of this dramatistic novel but also the basis for a possible interpretation of its gender politics? Her embittered thoughts can easily be inserted into the syntagmatic series of opposing materialistic, idealistic, pragmatic or mystical “casuistries” of the novel – with Karin Michelson appearing not only as a representative of the last option, but above all as the only character who explicitly denounces Niels Nielsen’s deep seated convictions as sheer “casuistry” and empty rhetoric ( 5 on page 247). She thus embodies the position of the woman as an outsider to civilisation, of someone who, according to Freud, is simply forced to adopt a hostile attitude towards it. Her main dramaturgical function – as an object of desire who unites the majority of other male characters in the novel in a mimetic chain of mutual rivalry and imitation – therefore rather puts her character into the paradigmatic series of mutual substitution, in which Karin is linked to and replaceable by other female characters: not only Nielsen’s mother, Solvejg, and the two estranged wives of the two former school friends, but also by allegorical emblems of Blithuania itself. This paradigmatic, vicarious logic of the novel, which largely functions beneath or beyond the represented crude *Realpolitik* of the country, simultaneously provides the most prominent and yet largely unconscious motivation for the concrete actions of the novel’s male characters. But it reveals itself only in the oneiric images that haunt both Barutanski and Nielsen in the third book, as a reminder that the two men are doubles of each other, as they are participants and mutual mediators in what René Girard in his *Mensonge romantique et vérité romanesque*, that is, *Desire, deceit and the novel*, calls “the triangular desire” ^17^. Being an unconscious mechanism of identification and rivalry, it is here transposed to the overt ground of state politics, and the ever-intensifying cycle of violence that mimetic desire generates. What both Barutanski and Nielsen crave, then, is what they both simultaneously destroy: the human substance of Blithuania, the declared but in fact annihilated “purpose” in whose name they presumably fight their political battles.

That this human substance, this fundamental value, is gendered feminine is no great surprise: Krleža’s entire dramatic oeuvre, as Dževad Karahasan has pointed out using the example of the Glembays trilogy ^18^, is permeated by precisely such a triangular logic, in which women generally function as victimised and mystified objects of an exchange from man to man, or, to quote Krleža again, “as a kind of flesh,” “used young flesh”. Karin Michelson’s fate is just one of the variants of this mode of operation, which makes it clear that in *Banquet in Blithuania* the personal and political realms are coextensive and congruent. But the author ostentatiously kills her off quickly and has her transformed into the haunting oneiric image that is retroactively enriched in Nielsen’s remorseful conscience by the fetishistic embellishments of a mysterious *femme fatale* – a figure doomed to die in a political “film noir,” or rather, “roman noir”. Given this bend in perspective, which makes *femme fatale* the figment of Niels Nielsen’s imagination, it would be somewhat unfair to criticise Krleža for his use of this problematic figure, which has fared quite ambivalently in feminist discussions, ranging from the champion of female agency who defies the constraints of female conduct to a commercialised female ideal whose presumed transgression is just another guise of conventionality. According to feminist critics like Julie Grossman, the very employment of the “femme fatale” figure is “a way of thinking about female agency that fills a social psychological need to contain female energy and sustain the status quo with regard to gender roles” ( 19 on page 133). From this perspective, *femme fatale*’s dependence on oppression means that she never meaningfully inserts herself in society, lurking instead outside or beneath society as a much-needed negative force and detractor.

To be true, Karina does not entirely fit this description: formerly promiscuous and sullied by reputation, like Alicia from Alfred Hitchock’s *Notorious*, and, like her, inhabiting an impossible position, being “the locus of the crisscrossing of various desires and duties that seem to conspire to her downfall” ( 20 on page 63), and ending as a suicide, she is rather “trapped into performing the role of *femme fatale*” which deprives her of the complexity of her personal history ( 19 on page 25, 42). Misread by Nielsen not only as a traitor and a whore, but also as such a mystified aesthetic construct, she thus, like Alicia, expresses “women’s experience of the contradiction in patriarchy” ( 20 on page 63). In a way, Nielsen is – like Devlin with respect to Alicia – willing to share her masochism, since he also becomes a passive spectator, but this time of his own obsessive oneiric staging of her suffering (17 on page 67). Yet, for all his intertextually overloaded and multi-layered criticism of the sacrificial role that women inevitably play in political transactions and sexual rivalries between men, Krleža seems here to endorse the viewpoint on the fateful origins of civilisation in the expulsion of women and repudiation of femininity that was propounded by psychoanalysis and anthropology in the mid-20 ^th^ century.

 The same radical insight into “the scapegoat psychology of the gender system, war and fascism” ( 10 on page 289) is at the basis of Virginia Woolf’s *Three Guineas*, her most controversial feminist essay, written shortly before the novel *Between the Acts*. In the novel, as Christine Froula suggests, the origins of this psychology in the ‘master plot of civilisation,’ as outlined by Freud’s psychoanalytic and Girard’s anthropological writings, are further explored, but with a view to possible revisions. Refusing the identification of woman with the nature that civilisation necessarily opposes, the novel, according to Froula, presents the civilisation as “unnaturally masculine,” framing instead “woman as civilisation’s natural inheritor, an insider surprised to find herself forced outside,” who “looks beyond brute force to the reason and speech that distinguish the human species and lays claim to civilisation” ( 10 on page 294). Shocked by the news reporting on a gang rape, and impatient with the obscurity of female authorship – symbolised by the female director, Miss La Trobe, hiding herself from the audience – the protagonist of the novel Isa, who is not only a wife and a mother, but also a secret poet of sorts, feels that “it is time someone invented a new plot, or that the author came out from the bushes” ( 2 on page 215).

 Unlike Krleža, who sets his premiere of the ambitious, male-written avant-garde play entitled *Puppets* in a provincial Blatvian *Burgtheater* and has it act as a trigger for Niels Nielsen’s solipsistic, guilt-ridden daydreams, Woolf does something that is both parallel and methodologically opposite: she imagines a ridiculous open-air historical pageant in the countryside, written and directed by a woman of uncertain national origin and sexual preference, and offered to a whole series of spectators who struggle to form “an intermittent we from disparate I’s” ( 10 on page 289), divided as they are along gender lines into “unifiers,” like Lucy, and “separatists,” like her brother Barth. If Krleža alludes to his own avant-garde phase in the above-mentioned performance, it is because he is concerned with the uses and abuses of art in unbearable political circumstances, with senseless regional rivalries and with the political “puppetry” of the small nation dancing to the tune of the grand plan of international economic and colonial interests, but his main concern remain the pitfalls of individual male conscience and of individual political resistance. Having gone through his trials and tribulations or, as Krleža puts it, his “moral and political catharsis,” Nielsen seems to follow Fortunato Yorick’s exhortations at the end of the novel by finally joining the revolutionaries who have executed the earlier clique, despite Karina’s corpse weighing on his conscience, and despite her warnings that love for her is the ultimate loyalty, that it means sharing the moment of the other’s death without hesitation. I say “seems” because the novel draws to a close with Nielsen acting as if “under anaesthetic” ( 5 on page 338) and with his disquieting suspicion that his return to Blithuania is again a puppet-like response to a futile call of his tormented conscience, since “this kind of silly sabre-rattling on the political stage serves no sensible purpose” ( 5 on page 340).

However, with her scattered and failed pageant, which depicts selected episodes from British history in a style that imitates or alludes to well-known writers from Chaucer via Shakespeare to Congreve, and presents scenes that mostly revolve around typical romantic plots, Woolf aims for a more politically durable common ground. She is more interested in the feelings and thoughts of the community that the play addresses and reassembles rather than in the individual consciousness and its choices, whether artistic or mundane. While she is primarily poking fun at the English imperial legacy, rooted in male cultural self-assurance and epitomised in the character of Giles, the heir to the Pointz Hall estate where the pageant is set, she also pokes fun at her own doppelgängers in the novel – Lucy Swithin and her mystical longing to go back to prehistory to enjoy her “moments of being” in the quiet depths of her mind and imagine an alternative to the corrupting course of civilisation, as well as Miss La Trobe and her experimental aspirations as a playwright and director, which are in no uncertain terms likened to artistic dictatorship. The fact that the performance is exposed to the outside world, so that at times it collapses under the pressure of natural elements such as wind, rain and the mooing of cows, is a sarcastic Woolfian allusion both to Miss La Trobe’s and her own literary dissatisfactions, and to the negotiations between nature and patriarchal culture, which can only re-establish its supremacy through weapons – the twelve aeroplanes that herald war.

But the most significant aspect of the novel’s highly deliberate gender politics lies in its multifaceted and complex, conflicted treatment of what Woolf, in her novel *Jacob’s Room*, while referring to the ultimate unknowability of the other, calls “the conditions of our love” ( 21 on page 116) – be it the love between the dour Bart, of the Indian Civil service, and the ever-curious, spiritual Lucy, an older brother-sister pair; the love between the melancholy, even suicidal Isa and the action-driven, frustrated stock-broker Giles, a married couple; the love between Isa, the tender mother, and her lively young son George; the love between friends, the “wild child” Mrs Manresa and the homosexual William Dodge; or the love between illicit lovers who either exchange fleeting flirtations, like Isa and William Dodge, and Isa and her neighbour, the farmer Mr. Haines, or have actual clandestine sexual encounters, like Giles and the socially and sexually overbearing Mrs. Manresa. All these orthodox and unorthodox couples, regardless of the diversity of their age and sexual identifications, are, as Isa says, “torn asunder” by “love and hate” ( 2 on page 215), since they all seem to be affected by the constraints of patriarchy and therefore forced to play predetermined, heteronormative gender roles, prefigured by the two historical portraits hanging in Pointz Hall, of a distinguished male ancestor, and a mysterious lady whose history remains unrecorded, as well as by the insistent popular rhyme “The King is in his counting house, counting out his money, the Queen is in her parlour, eating bread and honey” ( 2 on page 122). All characters therefore lie and cheat in various ways, suppressing, like Isa, their innermost aspirations and feelings of loneliness while lethargically going through the motions in a futile search for communication and intimacy. Even when confronted with Miss La Trobe’s satire on Elizabethan national symbols, on the 18 ^th ^century’s cult of reason, on the self-righteousness of colonial superiority, on idealised Victorian constructions of home and marital bliss, and not least on their own petty appetites for lust, money and power, they remain the same incoherent “orts, scraps, and fragments” ( 2 on page 215) embodied and reflected by the fragments of broken mirrors held by the amateur actors in the final scene of the pageant. For all the freedom they are given during and between the acts of the performance to co-create, to talk, judge and socialise, they seem unable to grasp the meaning of the play and to recognise their own contribution to the logic of patriarchal civilisation, which favours their competitive and violent instincts and thus inevitably leads to war. The same kind of stubborn refusal to see plagues Nielsen’s tortured mind when he bemoans “the Blitvinian lack of understanding for even the most insignificant order and patterns of phenomena,” but he is quick to attribute this to the scene he is, like Hamlet “born to set right,” i. e. “the wretched circumstances in that wretched country of winds, rain, and bedbugs” ( 1 on page 91). Of course, the idyllic English countryside offers no such excuses.

Although the spectators of Miss La Trobe’s pageant remain distracted and seem to have little chance of evolving, let alone of reshaping the plot, the novel itself creates a common ground for communal polyvocality, for differences and disagreements as seeds of an eventual transformation of all theatregoers into true members of the public realm. It is difficult to derive a definitive understanding of Miss La Trobe’s experimental endeavour from their scattered comments, especially as Woolf is keen to point out their different gender identities and class and educational backgrounds. However, it is through the juxtaposition of their contradictory statements about Miss La Trobe’s intentions, the boundaries of fiction and reality, and the role of the audience in the completion of the artwork that the novel achieves its own narrative – or should I say dramatistic – experiment in staging the risks and rewards of political dissensus, to use Jacques Rancière’s concept ^22^. Woolf’s ending could thus be interpreted as at least hinting at the possibility of a new plot, provided that the woman and the man, Isa and Giles, after their inevitable ‘fights,’ engage in what Kenneth Burke would call ‘true collaboration,’ an equal and truly loving dialogue, the kind of dialogue perhaps suggested by Luce Irigaray in her *La voie de l’amour*, *The Way of Love*, since in this essay she also imagines a couple in love, sharing a new, non-appropriative language, a language attuned to the irreducibility of the other and letting the other be silent before responding, a utopian “language of exchange between cultures, traditions, sexes, generations” ( 23 on page 42).

The final scene of *Between the Acts*, which takes place between Isa and Giles, is announced with words that are just as theatrical as those with which Krleža began: “The curtain rose. They spoke” ( 2 on page 219). The imaginary prospect of Krleža’s *Comoedia Blithuanica* is that it continues in the same old cauchemaresque manner, with Nielsen as the persistent journalistic or literary chronicler of its course. Given the melancholic tone, and the gaps and silences that pervade *Between the Acts*, its jarring rhythm, if not its verbal dance on the verge of the collapse of the entire symbolic order ^24^, the fact that Woolf’s narrator does not resort to the most notorious of Shakespearean allusions at the end and does not proclaim that “the rest is silence” is perhaps the best comfort the reader can take from Woolf’s equally daunting, ambiguous final intimation of an unknown and uncertain future awaiting humanity.

## Ethics and consent

Ethical approval and consent were not required.

## Data Availability

No data associated with this article.

## Footnotes

^1^ This is the title as it was translated in English by Edward Dennis Goy and Jasna Levinger-Goy for Northwestern University Press edition ^1^. I would however prefer *Banquet in Blithuania*, since the allusion to Lithuania is quite intentional.

^2^ I refer to the following edition: Virginia Woolf, *Between the Acts* ^2^.

^3^ For all references to the third book, I will use the following edition: Miroslav Krleža, *Banket u Blitvi* III ^5^.
