# Supplementary material for: Love versus/and/as politics in Miroslav Krleža’s Banquet in Blitva and Virginia Woolf’s Between the Acts
Source: Open Res Eur. 2025 Jul 23;5:189. [Version 1] doi: 10.12688/openreseurope.20783.1 (PMC12933038; doi:10.12688/openreseurope.20783.1)
Supplement: Peer review history

# Reviewer report: Reviewer response for version 1

**DOI:** 10.21956/openreseurope.22487.r69045

**Published:** 2026-02-24

**Authors:** Miranda Levanat Peričić

**Affiliations:** 1 University of Zadar, Zadar, Croatia

The paper provides a comprehensive comparative study of two novels: Miroslav Krleža’s  three-volume *Banquet in Blitva* and Virginia Woolf’s posthumously published *Between the Acts*. Written during the pre-war period on the eve of World War II, both novels undoubtedly belong to the canon of the 20th-century political novel. The author of this study bases her comparative reading on the parallels she identifies in their primary compositional technique, asserting that it is grounded in theatrical frameworks and metaphors.

 The analogies with drama in both novels stem from the inclusion of a play-within-the-play in their composition. While the embedded drama in Woolf's novel contains typical romantic scenes and selected episodes from British history, in Krleža's novel features a puppet performance with a protagonist named Fortunato Yorick. This serves as a key argument for the author to interpret the function of this performance as a kind of “Mousetrap-like play-within-a-play-within-a-novel”.

 Furthermore, the author explores the “grammar of motives” in Krleža's novel through the interpretive lens of Kenneth Burke's dramatistic model. She argues that both Krleža's and Woolf's novels can be described as 'dramatistic' in the Burkean sense, as both utilize a theatrical framework to define the fundamental tensions and ambiguities within the Burkean pentad: act, scene, agent, agency, and purpose.

 The genre implications of this situation are further stimulated by Krleža’s own assertion that the center of his novel is not a political but a love plot. This prompts the author to reach the bold conclusion that the core of the novel is, in fact, a psychological drama. To support this, she focuses on the dramatized relationships between the characters, examining them not only through the contrast of their political discourses but also through behavioral patterns marked by gender politics. In this regard, the author also interprets pre-existing drama as a potential framework for the dramatization of Krleža's novel.

 From the outset, this work adopts a critical stance toward prevailing readings of Krleža's novel, particularly regarding the role and type of the narrator. Specifically, the author challenges the predominant view of an authoritarian narrator, proposing instead a thesis of polyphonic structure. She supports her claims with exhaustive analysis and convincing, cogently presented argumentation. Finally, by focusing on the role of Karin Michelson in the third volume of the novel – where she emerges as a central figure haunting the protagonist's unconscious while embodying the position of women as outsiders of civilization – the author offers a highly original reinterpretation of the ‘love theme’. Ultimately, this study transcends traditional scholarship by positioning the romantic plot not merely as a subplot, but as the very dramaturgical core and the essential foundation for a nuanced understanding of Krleža’s gender politics.  Such an approach provides a fresh and significant contribution to the comparative study of modernist political prose.

 The reviewer's only minor remarks concern the formatting of the bibliography. While the list complies with the publication's requirements, it would be clearer if it were organized in alphabetical order. Furthermore, there appear to be omissions in the references, as not all works cited in the text (e.g. Žmegač 2001 and Kovačević 2013) are included in the final bibliography.

If any, are all the source data and materials underlying the results available?

Partly

Is the work original in terms of material and argument?

Yes

Is the work clearly and cogently presented?

Yes

Is the argument persuasive and supported by evidence?

Yes

Does the research article contribute to the cultural, historical, social understanding of the field?

Yes

Does it sufficiently engage with relevant methodologies and secondary literature on the topic?

Yes

Reviewer Expertise:

Comparative literature, genre theory, narratology, post-Yugoslav literature, posthumanism and novel

I confirm that I have read this submission and believe that I have an appropriate level of expertise to confirm that it is of an acceptable scientific standard.

# Reviewer report: Reviewer response for version 1

**DOI:** 10.21956/openreseurope.22487.r69044

**Published:** 2026-02-09

**Authors:** Renata Jambrešić Kirin

**Affiliations:** 1 Institute of Ethnology and Folklore Research, Šubićeva, Croatia

In addition to being original in terms of new, unexpected and analytically masterful comparison, Lada Čale Feldman’s article “Love versus/and/as politics in Miroslav Krleža’s *Banquet in Blitva* and Virginia Woolf’s *Between the Acts” *introduces one of the greatest, yet insufficiently translated, Croatian writers—the leading figure of Croatian modernism, Miroslav Krleža, to an international public. The main arguments are clearly and coherently stated, showing why a comparative analysis of two novels written in the late 1930s and received primarily after World War II is justified. The author demonstrates that these novels are closely related in many aspects and that the recent interests in gender politics of writing and in the political agency of literature could and should return them to the focus of both general and scholarly readerships.

 Having pointed out that the number of critical responses to Miroslav Krleža in a European context cannot be compared to those enjoyed by Virginia Woolf, Čale Feldman approaches both novels with equal insightfulness, sensitivity, and outstanding analytical acuity. Her preference for feminist literary criticism in no way implies a preference for *Between the Acts*. It is also worth noting that Krleža’s three-volume anti-militarist novel *Banquet in Blitva* was translated (partially) into English only in 2004, and I believe that this article will further stimulate the interest of literary scholars in the oeuvre of distinguished Croatian writer.

 The author’s principal aim—to describe unexpected literary affinities and parallels, that is, “formal, thematic and contextual correspondences,” between *Between the Acts* by Virginia Woolf and *Banquet in Blitva* by Miroslav Krleža—is fully achieved. Beyond the structural correspondences in the construction of these two multi-layered, polyphonic fictional worlds, Čale Feldman places particular emphasis on the intellectual breadth and ethical concerns shared by both writers: their preoccupation with the rise of European fascism and dictatorial regimes in the late 1930s, their anxiety over the “uses and abuses of art in unbearable political circumstances,” and their sustaining faith in the power of the literary word—especially in the capacity of theatre to exert moral and affective influence on its audience.

 The article uncovers the central “grammar of motives” (Kenneth Burke) in both novels, elucidating their core narrative strategies and rhetorical devices, the function of the narrators’ (polyphonic) voice, stylistic features, and the symbolism of characters divided along gender, social, and ideological lines. It also explains the inherent ways in which these exceptional novels resist becoming overt discursive arenas for cultural and political ideas. Drawing on the analytical concepts of Kenneth Burke and René Girard, and especially on feminist literary critics such as Christine Froula, Elisabeth Weiser, Julie Grossman, and Anna Kornbluh, Čale Feldman persuasively demonstrates that the resonant points of both novels emerge in their treatment of the interdependence of fascism, patriarchy, and militarism; in their exploration of the relationship between culture and politics, human nature and civilization; in the introduction of “internal theatre frames and theatrical metaphors”; in authors’ inclination toward irony and sharp humor; and in their effort to balance poetic experimentation with politically engaged “cautionary literature.”

 The differences between the two fictional worlds are articulated even more clearly—and, I would say, with greater feminist passion, expertise, and theoretical investment. Through a feminist reading of René Girard’s concept of “triangular desire,” Čale Feldman shows how, in Krleža’s work, male protagonists activate an “unconscious mechanism of identification and rivalry,” including an “ever-intensifying cycle of violence that mimetic desire generates.” At the same time, female characters—regardless of their contrasting psychological personae—“generally function as victimised and mystified objects of an exchange from man to man.” Although the male protagonists represent conflicting ideologies and worldviews, from the perspective of mimetic desire they reproduce a masculine principle of struggle for power, female affection, and prestige, while ruthlessly destroying the lives of ordinary people and the “human substance” of Blithuania, which bears the traits of a “gendered feminine”: social vulnerability, precarity, and instability that undermine the myth of Western civilizational progress.

 By contrast, the author demonstrates how Woolf’s *Between the Acts* offers complex portrayals of love and gender politics in multiple forms—familial, marital, friendly, and illicit—while also revealing the resilience of patriarchal structures that impose rigid, heteronormative gender roles. Woolf employs a proto-theatrical, satirical pageant through which her alter ego critiques historical myths, nationalism, colonialism, and ideals of domestic harmony, yet fails to awaken its (provincial) audience to their complicity in these patriarchal and rigid systems of social reproduction. Despite this failure, the novel’s central proto-theatrical scene creates space for political and social transformation by staging multiple and conflicting perspectives among the pageant’s spectators. Through her ambiguous interpretations of the scene, Woolf enacts a form of productive dissensus that gestures toward the possibility of change, new artistic forms and new social constellations.

 In conclusion, I assess this well-structured, persuasive and cogently presented article a theoretically and culturally relevant scholarly work—one that affirms the value of the comparative method, close reading, performative theory, and feminist literary criticism while simultaneously contributing to a better cultural, historical and ideological understanding of two distant European literary fields.

If any, are all the source data and materials underlying the results available?

Yes

Is the work original in terms of material and argument?

Yes

Is the work clearly and cogently presented?

Yes

Is the argument persuasive and supported by evidence?

Yes

Does the research article contribute to the cultural, historical, social understanding of the field?

Yes

Does it sufficiently engage with relevant methodologies and secondary literature on the topic?

Yes

Reviewer Expertise:

Comparative literature, Gender politics in the XX century, Women's history

I confirm that I have read this submission and believe that I have an appropriate level of expertise to confirm that it is of an acceptable scientific standard.
